# Synergistic Effects of Climate Change and Marine Pollution: An Overlooked Interaction in Coastal and Estuarine Areas

**DOI:** 10.3390/ijerph16152737

**Published:** 2019-07-31

**Authors:** Henrique Cabral, Vanessa Fonseca, Tânia Sousa, Miguel Costa Leal

**Affiliations:** 1 Irstea, UR EABX, Centre de Bordeaux, 50 avenue de Verdun, 33612 Cestas, France; 2 MARE – Marine and Environmental Sciences Centre, Faculdade de Ciências da Universidade de Lisboa, Campo Grande, 1749-016 Lisboa, Portugal; 3 MARETEC - Marine, Environment and Technology Center, Department of Mechanical Engineering, Instituto Superior Técnico, Universidade de Lisboa, 1049-001 Lisboa, Portugal; 4 Departamento de Biologia & CESAM, Universidade de Aveiro, Campus Universitário de Santiago, 3810-193 Aveiro, Portugal

**Keywords:** contaminants, global change, synergistic effects, coastal zones, literature review, methodological approaches, interactions, biological effects

## Abstract

Coastal areas have been increasingly affected by human activities, marine pollution and climate change are among the most important pressures affecting these environments. Human-induced pressures occur in a cumulative way and generate additive, antagonistic or synergistic effects. Knowledge on synergistic effects is crucial to coastal zone management, since they may imply a change in human uses of these systems, as well as dedicated action plans in order to reduce hazards and environmental risks. In this work, we provide an overview of the available literature on synergistic effects between climate change and chemical pollution, and discuss current knowledge, methodological approaches, and research gaps and needs. Interactions between these two pressures may be climate change dominant (climate change leads to an increase in contaminant exposure or toxicity) or contaminant-dominant (chemical exposure leads to an increase in climate change susceptibility), but the mechanistic drivers of such processes are not well known. Results from a few meta-analyses studies and reviews showed that synergistic interactions tend to be more frequent compared to additive and antagonistic ones. However, most of the studies are individual-based and assess the cumulative effects of a few contaminants individually in laboratory settings together with few climate variables, particularly temperature and pH. Nevertheless, a wide diversity of contaminants have already been individually tested, spanning from metals, persistent organic pollutants and, more recently, emergent pollutants. Population and community based approaches are less frequent but have generated very interesting and more holistic perspectives. Methodological approaches are quite diverse, from laboratory studies to mesocosm and field studies, or based on statistical or modelling tools, each with their own potential and limitations. More holistic comparisons integrating several pressures and their combinations and a multitude of habitats, taxa, life-stages, among others, are needed, as well as insights from meta-analyses and systematic reviews.

## 1. Introduction

Coastal areas comprise a wide diversity of ecosystems (e.g., estuaries, coral and other biogenic reefs, sandy and rocky subtidal areas, mudflats, mangroves, saltmarshes, eelgrass beds), provide multiple ecosystem services, and have a high ecological and economic value [1,2]. Human activities have been intensively affecting these areas for many decades, thereby inducing profound changes in their structure and functioning. In a global assessment of the health of marine ecosystems, Halpern et al. [3] outlined that no area was unaffected by human influence and that more than 40% of marine areas were strongly impacted by multiple pressures. The magnitude of human impacts has been higher in coastal zones compared to offshore areas, with climate change, fishing, and pollution being the most significant pressures [3,4].

Climate changes drive diverse impacts on coastal zones. There is evidence that the sea surface temperature (SST) has been increasing since the end of the XIX century [5], at an annual rate of about 0.11 °C from 1970 onwards, although the inter-annual variation was extremely high. In more recent periods, increases in the SST in coastal zones worldwide have been documented (e.g., [6,7]), being the estimates for the end of the XXIst century even higher than average for the global ocean (0.6 to 2 °C compared to estimates up to 4 °C in certain coastal zones) [5,8]. Some of the shallow or intertidal coastal habitats, such as saltmarshes, mangroves, and tidal flats, will be particularly exposed to extreme events like heat (or cold) waves, which will probably further increase the thermal stress in these environments. Global warming interferes directly with organisms but have also other effects on coastal processes. The volume of the ocean increased due to its thermal energy and due to the melting of ice caps that induced sea-level rise. Estimates of the mean sea level rise of 3 mm year^-1^ have been proposed, implying an increase of about 40 to 75 cm by 2100 [5]. Ocean acidification is also a human-induced major driver, resulting from increasing CO_2_ concentration in the atmosphere, and with impacts on a wide diversity of organisms, namely corals, planktonic organisms, and calcifying structure’s organisms. Estimates for 2100 indicate a decrease in 0.3 to 0.4 pH units [5,9]. Expected climate conditions also include an increase in the frequency of extreme events, especially storm surges, as well as changes in the water cycle dynamics that, depending on the geographical area, may be expressed as a strong reduction in rainfall and, consequently in drought events, or an intensification of rainfall during short periods, which also induce a severe regime shift [6].

Overexploitation of marine resources is also a major human pressure in marine ecosystems, especially coastal environments since these areas are highly productive and serve as nursery grounds for a wide diversity of species. Catches from fisheries peaked in late 1980s and have been relatively stable since then, at values around 90 million tons per year [10]. As a consequence of an increasing fishing pressure, marine fisheries resources have been declining worldwide. The percentage of marine resources’ stocks within biologically sustainable levels decreased from 90%, in the 1970s, down to 67%, in 2015, and the percentage of underfished stocks also decreased from 40%, in the 1970s, to less than 10%, in 2015 [10]. Coastal fisheries, that are mainly small-scale, represent more than 50% of global marine fisheries, while its area of operation corresponds to about 3% of the global ocean [11]. Besides the direct impact on the populations of harvested species (e.g., reduction in number of individuals, reduction of individuals mean length, earlier maturation and at smaller sizes; [12,13,14,15,16]), fisheries have several other effects such as bycatches and discards, ghost fishing, decrease in trophic level, bottom destruction, among others (e.g., [12,17]). Projections regarding fisheries trends and impacts often take into account foreseen climate changes. According to several authors, projected fisheries catches for 2050 may vary less than 10% [18,19], but a high spatial variation may occur, with a stronger decline expected for tropical areas [18].

Coastal ecosystems are also heavily affected by pollution. Human activities have introduced thousands of substances and/or materials in the marine environment that once above certain threshold values might present negative effects on biological components of these ecosystems and, therefore, become pollutants [20]. Pollutants might originate from a large number of human activities (e.g., agriculture, industrial, urban and port development, transportation, fisheries and aquaculture, recreation) and may be of a different nature—chemical (e.g., nutrients, biocides, metals, oil, pharmaceuticals), physical (e.g., plastic debris, large hard structures, temperature, radiation, noise), or biological (e.g., introduced non-indigenous species). The classification of pollutants is not, however, straightforward, since a certain element may be toxic (i.e., that can be poisonous or have negative physiological effects) and simultaneously introduce physical and biological changes (for example, a toxic plastic debris colonized by non-indigenous species). Pollutants may be introduced in marine coastal areas directly as discharges or solid wastes from land and human activities at sea, through the runoff of rivers (these being point-sources for the marine environment), or by exchanges with the atmosphere (non-point-source) [21]. The relative contribution of these three pathways varies according to the type of pollutant, and its estimation is extremely difficult and uncertain due to the high spatial and temporal variation and to the lack of knowledge of several processes determining their exchanges and fate [21]. Furthermore, pollutants incorporate biogeochemical cycles in these coastal areas, which introduce a high complexity to their quantification and their impacts assessment. Trends in coastal pollution are also extremely variable. While for certain geographical areas successful efforts have been made in order to reduce some pollutants (e.g., polychlorinated biphenyls in the Baltic [22]; pesticides in the Caribbean Sea [23]), in other zones and/or other contaminants increased pollution has been reported [24]. Nevertheless, several authors are very cautiousness in predictions relative to changes in coastal pollution, since it may be a result of the economic and technological development, for which future developments are difficult to predict [24]. Among other pressures affecting coastal zones, several authors emphasize the relevance of habitat loss and non-indigenous species, with their magnitude being extremely different according to geographical area, although with documented impacts worldwide [3,25].

For the large majority of coastal zones, human pressures occur in a cumulative way, interacting with each other and generating effects that may be additive, antagonistic or synergistic [26]. Despite the increasing volume of literature addressing cumulative impacts, there is a huge gap in the knowledge on how different pressures may interact, what are their resulting effects, and what conservation and management decisions should be made to protect and restore marine coastal ecosystems. In this review, we evaluated the synergistic effects of two of the most important pressure in coastal zones—chemical pollution and climate change. We critically review the available literature and discuss the current knowledge, the main methodological approaches that have been used to evaluate them, and also identify the main research gaps.

## 2. Marine Pollutants in Coastal Areas

Marine pollutants comprise a wide diversity of physical, chemical, and biological agents that induce negative effects on ecological systems whenever their magnitude is above a certain threshold. There are many thousands of these agents that have been added or created by human processes, and for most of which, the available knowledge is very scarce, especially on their environmental and ecosystem impacts [24]. Agriculture, coastal tourism and recreation, port and harbor activities, urban and industrial development, mining, fisheries, and aquaculture, are all sources of marine pollution threatening coastal and marine habitats [24,27]. Pollutants, regardless of being physical, chemical, or biological, may be ocean-born or may reach marine ecosystems through rivers drainage, point sources, and atmosphere transfers. Despite the multiple origin of pollutants, river basins’ drainage and direct point sources are often the main pathways of land-based sources, which represent approximately 80% of marine pollution globally [24,27].

### 2.1. Eutrophication

Coastal eutrophication has dramatically increased in the past decades and is a widespread major problem that promotes several negative effects, among which hypoxia (concentrations of dissolved oxygen lower than 2 mg·L^−1^) is usually the most detrimental one [25,28]. Eutrophication is mainly driven by nitrogen inputs, although phosphorus has also a major role in this process [28,29]. Reactive nitrogen loads resulting from human activities, especially the use of fertilizers, agriculture of N-fixing crops, and fossil fuel combustion, have grown exponentially since the 1960s [30]. Despite the flux of nitrogen to coastal areas may be due to several processes, river basins input is usually the main contributor, which is in turn related to landscape use (especially agricultural use) and river flow regime [28]. Not surprisingly, the worldwide distribution of coastal eutrophic areas with an associated oxygen depletion match with major population centers and watersheds that have the largest inputs of nutrients [31,32]. In a global review on eutrophication, Diaz and Rosenberg [32] identified more than 400 coastal areas (covering more than 245,000 km²) for which hypoxia have been documented, it was found that its number has approximately doubled each decade since the 1960s. Regarding predictions on the evolution on coastal eutrophication, Howarth [28] outlined that since major sinks at the watershed and landscape levels are increasingly saturated, an increase in the input to coastal areas should be expected in the future. Nevertheless, a high level of uncertainty is still embedded in predictive modelling methodologies. According to Diaz and Rosenberg [32], hypoxia and anoxia are among the most widespread deleterious anthropogenic influences on estuarine and marine environments due to eutrophication. These authors further outline that dissolved oxygen in these environments have changed drastically over a short time, and that further expansion of hypoxic areas will largely depend on climate-related processes, namely water-column stratification and water basins runoff processes.

### 2.2. Metals

Metals comprise a large number of elements and derivate substances that occur in nature, usually in very small concentrations. This group is poorly defined, since it often integrates other trace elements that are non-metallic, as is the case of arsenic or selenium [33]. Human activities have increased drastically metals’ concentrations in the environment, and in coastal zones in particular, with industrial and mining activities being the main anthropogenic sources of metals [31]. Estuaries are often repositories or sinks of historical metal contamination associated with metals’ strong particle reactivity with sediments [34]. Disentangling the anthropogenic input from the natural background is a considerable challenge, particularly considering the multiple factors influencing particle formation and adsorption or desorption of metals in coastal environments, and hence their bioavailability.

Some metals are essential for biological processes while others are not metabolized, but in both cases they tend to be of high toxicity even in small concentrations [31]. Toxic effects of metal exposure encompasses an increased energy demand, which affects marine organisms’ metabolism and growth [35,36]. The toxicity of metals might also act as potent immunosupressors [37] or lead to impaired development and reproduction [38]. Their negative effects on biota are quite diverse and depend on metal concentration, its speciation, interactions at receptors sites, uptake into the organism, detoxification ability, among others [39,40]. As they are not degradable, they tend to accumulate in organisms throughout food webs and are especially critical for top predators [41]. A typical example is mercury bioaccumulation and biomagnification in marine food webs [42], given that mercury exposure has been shown to cause severe neurotoxic effects in marine fauna and humans [43,44]. Despite the reducing anthropogenic emissions observed in the last decades [45], mercury pollution is acknowledged as an issue worldwide due to its long-distance transport and persistence in the environment.

Most of the available literature regarding concentrations of metals in coastal ecosystems is relative to zinc, copper, chromium, lead, nickel, mercury, and cadmium. Although the comparison between different zones and periods is usually not possible due to the diversity of analytical procedures, coastal areas in Europe and Asia have shown the highest concentrations [31].

### 2.3. Organic Compounds

Organic pollutants comprise a huge variety of chemical species including both natural and synthetic substances [46]. Most of them present a high structural stability and bioaccumulation potential, and a long-range transport, and therefore often denominated persistent organic pollutants [31]. These pollutants originate in a wide diversity of human activities, namely the ones related to pest control in agriculture, pharmaceuticals, detergents, flame retardants, combustion by-products, di-electric fluids, among many others [47]. Polycyclic aromatic hydrocarbons (PAH), polychlorinated biphenyls (PCB), polybromodiphenyl ethers (PBDE), organochlorine pesticides (OP), hexabromocyclododecanes (HBCD), Dechlorane Plus (DP) and perfluoralkyl substances (PFAS) are some examples of these pollutants [48]. The majority of these compounds are highly toxic to biota and produce a wide range of metabolic responses, which may severely hamper physiological performance and survival (some are carcinogenic and/or endocrine disruptors). Several studies on trophic cascades based on estimates of organic pollutants have allowed to infer impacts at population, community, and ecosystem levels, but information on these aspects is still scarce (e.g., [49,50]). In regards to spatial and temporal patterns of contamination by these chemicals, some European and Asian coastal areas have been reported as having the highest concentrations, although studies are still lacking for wide geographical areas [31]. The assessment of their temporal variation on the basis of several monitoring programs using marine organisms (e.g., mollusks, fish, sea birds and mammals) as bioindicators have shown that some compounds, namely dichlorodiphenyltrichloroethane (DDT), PCB, hydrogen cyanide (HCN), PBDE decreased their concentrations in recent years, mainly in response to regulations, whereas others, such as HBCD and PFAS, have increased [51].

### 2.4. Emerging Contaminants

Over the past two decades, the increasing concentration of pharmaceuticals and other personal care compounds (PPCP), together with their ubiquity in the aquatic environment and evidence of adverse biological effects linked to exposure, has led to their classification as emerging pollutants of priority concern [52]. Pharmaceutical compounds are designed to target specific metabolic and molecular pathways in both humans and animals (medical and veterinary use), which are generally evolutionary conserved [53], thus they may be highly potent pollutants by eliciting biological effects at very low-concentrations. Personal care products are used for improvement of the quality of life and include products such as cosmetics, UV blocker agents (sunscreens) or fragrances [54]. PPCP residues are continuously released into the aquatic environment, leading to chronic exposure even for relatively degradable compounds, thus are generally regarded as persistent or pseudo-persistent pollutants [55]. Arnold et al. [56] coined the term “Medicating the Environment” referencing the continuous released of PPCP into surface waters with almost no legal restrictions for discharge. The major source of PPCP residues input into the aquatic environment is inadequately treated wastewater effluents. However, other dispersal pathways include manufacturing facilities, waste disposal, aquaculture, and animal husbandry and horticulture along rivers and in coastal areas [56]. PPCP occurrence in the marine environment was first presumed to be negligible due to likely high dilution processes. However, recent works have indubitably demonstrated the pervasive nature of these compounds, with significant concentrations of PPCP residues in estuarine and marine areas (e.g., [57,58]), as well as in marine macroalgae, bivalves, and fish [59]. Geographically, available data on marine environments refers mostly to Europe, North America, and China, with incipient or absent information of Africa, Latin America or the Caribbean [60]. With global consumption and access to PPCP rising, it will inevitably lead to higher ambient concentrations and greater potential for bioaccumulation and hazardous effects in marine fauna.

Microplastics are also emerging contaminants for which a large number of studies have been recently published [61]. The physical impact of microplastics in marine environments and organisms have been documented, while their ability to contribute to chemical pollution have been less stressed [61]. Indeed, microplastics may sorb and accumulate pollutants due to their chemical properties, small size and large surface to volume ratio, and they generally have a large potential for dispersal even for areas with no major pollution loads [31,61].

Awareness regarding several substances resulting from high technology industries (especially nanoparticles such as titanium oxides) is also raising. Nevertheless, most of the studies conducted in marine ecosystems have stressed their negative impacts on filter-feeder organisms [62].

## 3. Climate Change Drivers and Their Impacts on Pollutants

Climate change encompasses multiple physical and chemical changes in ocean conditions, including temperature, pH, salinity, oxygen concentration, ice coverage, and currents. These changes are also altering contaminants’ behavior in the marine environment, affecting its worldwide distribution and toxicity potential, thus reshaping the environmental impacts of marine chemical pollutants [63,64]. Accordingly, changes in seawater temperature, salinity, pH, and other environmental parameters have been associated with changes in fate and global cycling of chemical pollutants (e.g., [65,66]), their bioaccumulation (e.g., [64,67,68]), bioavailability (e.g., [69]), and toxicity (e.g., [70]) to marine organisms. Notwithstanding, chemical exposure may also lead to increased inability of organisms to cope with shifting environmental conditions linked to climate change. Following Alava et al. [64] terminology, interactions between climate change and environmental chemicals can either be climate change-dominant (i.e., climate change leads to an increase in contaminant exposure or enhances organisms’ susceptibility to chemical toxicity), or contaminant-dominant (i.e., chemical exposure leads to an increase in climate change susceptibility). Resolving such complex interactions include discerning among antagonistic, additive or synergistic effects. Briefly, antagonistic effects describe an interaction where one stressor offsets the effect of the other; additive effects equal the sum of each stressor separate effects for the scenarios tested; and synergistic effects in which stressors interplay results in a combined effect greater than the sum of their individual effects [71]. A straightforward example is the impact of different climate changes or pollution stressors on the growth rate of a particular organism, which can equal the sum of the observed impacts of each driver alone (additive effects), it can present smaller (antagonistic effects), or greater outcomes regarding individual effects (synergistic effects). Przeslawski et al. [72] performed a meta-analysis of the effects of multi-stressor studies targeting early marine life stages and described higher prevalence of synergistic interactions (ca. 65%) than additive (17%) or antagonistic (17%) interactions. Available studies mainly focused on combinations of temperature, salinity and acidification drivers, whilst interaction between these factors and pollutants are scarce for most taxa.

Synergistic effects are likely to occur as multiple stressors impact coastal ecosystems (e.g., [25]), which has important implications for improved knowledge and management of the ecological and economic consequences of both chemical pollution and climate change. In a recent study, Ellis et al. [73] reported that multiplicative or synergistic effects were more common than additive interactions when assessing the responses of estuarine taxa to increased sedimentation, nutrients, and metal loadings associated with both increased urbanization and global changes in coastal environments.

Overall, coastal ecosystems may be particularly conspicuous to interactions between climate-change and chemical pollutants, due to the convergence of high levels of anthropogenic contamination resulting from present-day and historical discharges of multiple chemical compounds, with environmental changes predicted to be more pronounced and frequent in coastal areas than in the open ocean, such as hypoxia and decreased pH linked to increased freshwater runoff and nutrient discharge (e.g., [74]).

## 4. Upscaling Synergistic Effects of Pollution and Climate Change at Different Biological Levels

The impacts of climate change and pollution have been thoroughly explored but mostly as independent stressors [68,75,76]. However, the potential interactions between these stressors are still far from being clear [25,77,78]. This is mostly driven by the great diversity of stressor pairs that are likely to occur in marine coastal environments. Nevertheless, a growing number of studies assessing the interactive effect of multiple climate change and pollution stressors on marine organisms has been recently published [64,79,80]. It is also important to note that most studies use individual-based approaches [81], but there are also more holistic studies assessing community responses [82].

Individual-level studies are probably among the most common, due to the possibility to test the interactive effects of pollutants and future climate scenarios under experimental settings. Research on the consequences of multiple combined stressors has mostly focused on sub-organismal and organismal level impacts in order to show synergistic interactions [83].

Temperature is one of the most common stressors assessed in studies using a multiple stressor approach. One the one hand, this has been driven by the identification of global warming as an emerging threat to natural ecosystems, but also by the importance of temperature as a driver of organismal metabolic rate. Temperature increase leads to a concomitant increase of metabolic rate. As a consequence, food consumption rate also increases in order to meet the higher energetic demand. This opens a direct gateway for pollutants in food items to enter into and bioaccumulate in biological systems and promote synergistic harmful effects of temperature and other stressors [64]. For instance, temperature and metal exposure have been recurrently reported to synergistically interact to affect metabolic and immunological processes [84].

Besides temperature, ocean acidification has also been a driver tested in multiple-stressors studies. For instance, the immune response of the oyster *Crassostrea gigas* was affected by a synergistic effect of ocean acidification and cadmium pollution [85]. Additional results for this same species and stressors showed that ocean acidification notably aggravated the toxicity of cadmium, particularly ROS production and DNA damage of hemocytes, and also caused histopathological damage and apoptosis [81]. These results are in agreement with the findings of Su et al. [86] regarding the effect of persistent organic pollutants (POPs) and ocean acidification on the clam *Tefillarca granosa*, and also with the information reviewed by Ivanina and Sokolova [80]. This latter study supports the synergistic impact of ocean acidification and metals on fitness-related functions of marine organisms. However, the synergistic effects observed in the latter studies do not represent a standard response of marine organisms to ocean acidification and metal pollution. For instance, Dorey et al. [87] observed no additive or synergistic effects of acidification and copper on mortality of the sea urchin *Heliocidaris crassipina*.

The reproduction capacity of marine organisms can also be impaired by the synergistic effects of pollutants and increased water temperature, as recorded for the mussel *Mytilus galloprovincialis* [88]. This mussel species has often been used as a model organism for testing the effect of pollutants and climate change on marine benthic species [89]. For instance, Nardi et al. [69] and Nardi et al. [90] recorded synergistic effects of multiple stressors, such as ocean acidification, increased temperature and metal pollution, in physiological processes associated with bioaccumulation, detoxification and other biological processes. The type of effects and their magnitude can also vary among life stages. Humanes et al. [91] showed that the effect of warming and eutrophication affect fertilization of the tropical coral *Acropora tenuis* in an additive manner, whereas the effect on embryos deformities was synergistic.

It is also important to note that synergistic effects of climate change and pollution extend beyond consequences for marine animals. The combined effects of eutrophication and acidification have also been recorded for marine algae, particularly an accelerated growth of filamentous turfs [79,92]. Indeed, results reported by Russell et al. [92] show that the synergistic effect was 34% larger than the combined additive effect of each individual driver. The effect of eutrophication and climate warming has also been documented for macroalgae by Gouvêa et al. [93], who concluded that large-scale declines in macroalgal production, which previous studies identified as being driven by nitrogen limitation, might actually be driven by the synergistic effects of nitrogen limitation and elevated temperature.

Besides effects at the individual level, community level impacts are also extremely relevant yet notably challenging to empirically assess. Nevertheless, micro- or mesocosms experimental approaches are a valid option to study the interactive effects of climate change and pollution at the community and ecosystem level. For instance, Coelho et al. [82] assessed the response of a marine microbial community to anthropogenic pollutants under an ocean acidification scenario. Results showed that the role that microbes play in biogeochemical cycles might be affected by the interactive effects of these two drivers. Unfortunately, this review identified a notable gap in the scientific literature for community-based studies identifying synergistic effects of climate change and pollution in coastal marine communities.

The synergistic effects of climate change and pollution in coastal environments is likely driven by a multitude of stressors acting at the individual and community level [94]. For instance, and as previously mentioned, the toxicity of numerous pollutants is expected to be higher under increased temperature, and the concentration of pollutants driven by agricultural runoff is expected to increase with decreasing rainfall. In contrast, increasing rainfall and flooding driven by climate change might also deliver more pollutants from agricultural fields, plantations, and impervious surfaces, such as urban environments, parking lots, pavements, and roads, to the aquatic and coastal-marine environment [95,96]. However, the mechanistic drivers of such physico-chemical stressors at the organismal level are yet poorly understood.

Multiple elements have been recorded for individual-based and community-based experiments, and the data generated in such experiments have often been used for building predictive ecosystem models that take into account the multitude and complexity of species interactions [63,68,73]. Nevertheless, it is important to consider that well-known synergistic patterns, such as the synergistic toxicity effect of trace meals at higher temperatures [71], can show strong geographic signals that are driven by local adaptation mechanisms driving thermal and pollution tolerance [97]. This highlights that predicting the impact of different pollution drivers in a climate changing coastal environment can be extremely challenging due to the unique responses of different species, but also different populations locally adapted to a particular environment.

Statistical models used in ecosystem-level approaches allow research to address multiple stressors simultaneously. For instance, Ellis et al. [73] showed that sedimentation, eutrophication, and metal pollution synergistically drive biodiversity loss and affect ecosystem stability and functioning. One key result is that the synergistic effect of these stressors is greater on species diversity than on ecosystem traits. This study additionally highlights the importance of multiple-stressor studies, as single stressor studies overlook the interactive effect of different drivers, regardless of being antagonistic or synergistic. Another study has demonstrated the synergistic effect of eutrophication and climate change on estuarine and coastal waters, and has identified enhanced vertical stratification and periods of increased flushing time as the mechanistic drivers for an increasing frequency of harmful algal blooms [63].

Individual and community-based studies can also be used to infer ecosystem effects, particularly if the measured indicators refer to processes that drive ecosystem functioning, such as resource processing, decomposition rates, or other processes and services that directly quantify ecosystem functioning [83]. As an example, changes in grazer communities driven by multiple stressors can act synergistically with eutrophication, thereby promoting shifts in biodiversity levels and nutrient cycling [98]. Results from the latter study also highlight that loss of predators can play a critical role in the capacity of marine coastal ecosystems to respond to environmental changes combined with anthropogenic pollution.

## 5. Methodological Approaches to Address Synergistic Effects

The challenge to identify synergistic effects among climate change and pollution-related stressors is often associated with methodological limitations. While traditional ecotoxicological studies focused on the incremental effect of a pollutant on a particular biological organism, the complexity of experimental design, logistics, and data analysis of multiple-stressor studies notably increases with the number of stressors. Nevertheless, multiple approaches have been used for different levels of biological organization, such as experimental approaches for individual-based studies, to statistical modelling and trophic-dynamic ecosystem simulation model (e.g., Ecopath with Ecosim, EwE; see Reference [99]) approaches for studies at the ecosystem level.

Experimental approaches to assess the combined impact of multiple drivers not only require assessing the effects of such drivers, but also testing more than two scenarios for each targeted category. Resolving the interaction between stressors requires obtaining performance curves for each individual driver, together with a better understanding of their mechanisms or mode of action. However, such performance curves for each scenario require more than two levels for each stressor, regardless of the stressor being temperature, ocean acidification, or concentration of a chemical pollutant.

Laboratory studies allow testing individual and multiple stressors simultaneously and targeting specific endpoints or pathways to better understand how each stressor and their combination affect organisms’ responses. Although more ecologically realistic than single-factor studies, multifactorial studies may still oversimplify complex systems [72]. Laboratory studies can be viewed as a mechanistic approach to multi stressors testing, yet limited to organisms that can be kept in laboratory settings. Another limitation of these studies is their ecological relevance, given that they mainly focus on individual or sub-individual responses of model and a few non-model species, which can render different results. Furthermore, although differences in species’ sensitivities to ocean change stressors undoubtedly reflect different tolerance levels, inconsistent methodologies also play a significant part in different outcomes among studies [100].

Exposure time can also be controlled in laboratory studies. In contrast, exposure time is much more complex to control in the field, and it is difficult to guarantee that exposure occurs simultaneously with the stressor. A popular option includes sampling organisms from nature, in different locations and seasons, and exposing them to multiple stressors in laboratory settings [69]. Researchers using this approach must carefully design their experiments to guarantee the appropriate control treatments, since sampling, transportation, and husbandry in controlled settings are likely to cause stress to the organism of interest. Another approach to addressing multiple stressors’ interactions between climate-change and pollutants is mesocosms studies (e.g., [98,101]). They can be viewed as an intermediate design to laboratory and field-studies, or as a simplification of natural systems enabling mechanistic testing of single and multiple drivers in highly-replicable and controlled settings, and at ecologically relevant scales [102].

Field studies are also common, but identifying synergistic effects of climate change and pollution is a difficult challenge that often relies on powerful statistical tools [103,104]. Additionally, it is often difficult to define baseline reference points and thresholds, as there is no control treatment in field studies. Such information is typically determined from comparisons to historical data-sets or by sampling other locations that can be used as reference. Expert opinion might also be used to help define model assumptions and interpret results [105]. Note that ecosystem-based modelling requires a great multi-disciplinary work to guarantee that all ecological indicators are accurately sampled and interpreted in complex marine coastal ecosystems, such as estuaries, coral reefs, mangroves, among others. Ultimately, food web and trophic-dynamic ecosystem modelling, such as the Ecopath with Ecosism (EwE) model and the Ecotracer routine to track pollutant bioaccumulation under climate change forcing have also successfully been applied to assess and project simulations of the interactions of climate change impacts and pollutant bioaccumulation in the marine ecosystems [68,106]. These modelling tools have been used to model the bioaccumulation and biomagnification of pollutants in food webs, including global contaminants of concern, such as PCBs and methyl mercury.

**Figure 1:**
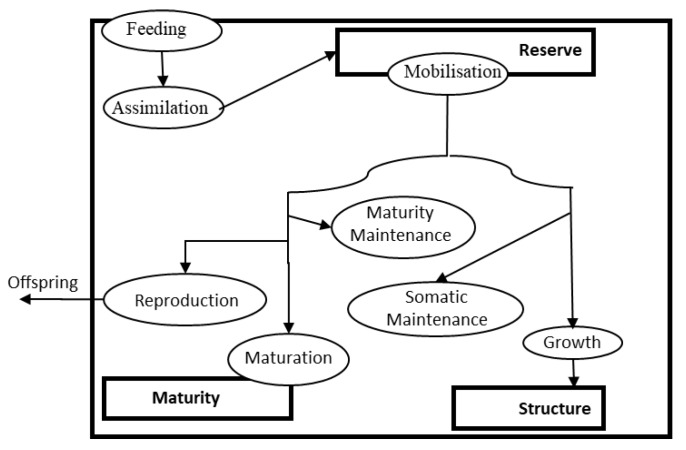
Scheme of the standard Dynamic Energy Budget model for an organism. Rectangles are state-variables reserve, structure, and maturity. Metabolic processes are ellipses.

Besides ecosystem-based modelling tools, a theoretical and modeling framework that has been gaining momentum for ecotoxicology and multiple-stressor studies is Dynamic Energy Budgets (DEB) [107,108,109,110]. DEB theory is a non-species specific theory at the organism level that describes and quantifies metabolic processes, such as the growth, development, and reproduction of organisms. The metabolic organization of the standard DEB model, which is applicable to animals that maintain their shape as they grow, is shown in Figure 1. The biomass of the organism is composed by structure and reserve while the life-stage that controls the metabolic processes that the organism has access to is controlled by the level of maturity. Organisms increase their maturity until achieving first the level of maturity that triggers the feeding behavior and then the level of maturity that triggers the reproduction behavior. The mass and energy allocated by the organism to metabolic processes such as growth and maintenance depends on the state of the organism (maturity, reserve, and structure), on environmental variables, such as the amount of food and temperature, and on species-specific parameters, such as the specific assimilation rate, the volume specific somatic maintenance rate and the specific volume cost of growth among others (for a more detailed description of the equations and the parameters used in DEB theory see [109]).

A first rough estimate of these species-specific parameters can be made using only the maximum length of the species and the scaling laws predicted by the theory. Estimation of these parameters, including the parameter that quantifies the dependence of rates on temperature, can be improved using published, field or laboratory data, such as growth curves and reproduction rates. Using these procedures, DEB species-specific parameters have now been estimated and are freely available for more than 1000 species [111,112]. Additionally, data is also needed to estimate the mode of action of a toxic compound on a particular species. Temperature affects metabolism through the parameters that are time-dependent while toxics affect metabolism through the parameter that is linked to the mode of action of that particular toxic. Thus, a DEB model can easily combine the effects of climate change and pollution to estimate growth and reproduction rates under conditions that are different from the field or laboratory experiments. DEB allows modelling the effects of multiple stressors, such as temperature and pollutants, among others [113,114]. Moreover, DEB can be applied to individuals to estimate how different stressors act at multiple biological organization levels, such as populations and ecosystems [115].

## 6. Knowledge Gaps and Future Prospects

**Figure 2:**
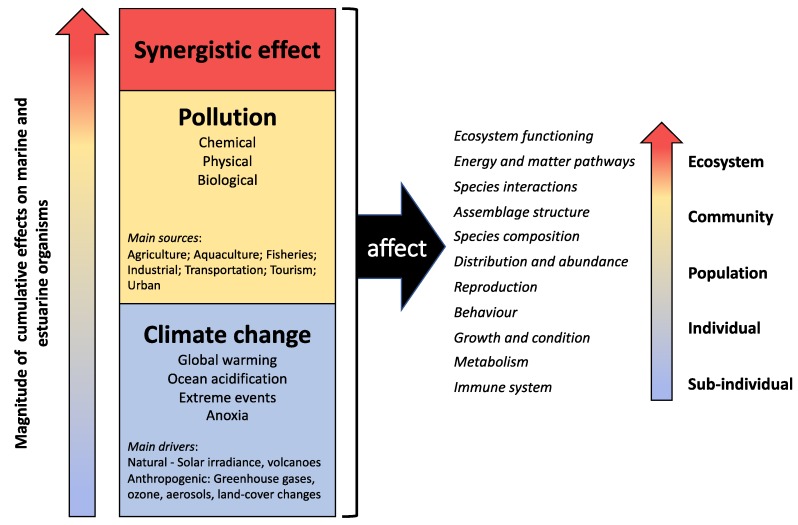
Conceptual model diagram of the cumulative and synergistic effects of climate change and pollution on marine coastal and estuarine.

Considering the huge diversity of contaminants, climate variables, and their combinations it is extremely challenging to have a complete overview of their interactions and combined effects (Figure 2). Furthermore, all methodological approaches have their limitations and potential, which increase the complexity of the studies aiming at evaluating synergistic effects. Niinemets et al. [116] stressed that the joint efforts of researchers working at different levels of biological organization are needed to understand and predict global change effects on various functional types of organisms and scale up from physiological responses to large-scale integrated ecosystem responses in future climates. All contributions, from individual- to ecosystem-based, and from laboratory to field studies have increased our knowledge and are often important to set the next generation of research priorities and set study designs. Coastal areas are probably the most important areas in the study of contaminants and climate change interactions, since it is in these environments that human pressures are more intense. However, comparisons with other marine ecosystems could also be of extreme value where some of these pressures could be isolated, or at least studied in a more restricted combination. Multi-site, multi-taxa, etc., studies have also an extremely high ecological relevance, as they enable comparisons between different habitats, species, and life-cycle stages, among others.

Future research should try to include more stressors in order to have a more holistic and comprehensive perspective of interactions, but in a more aggregated way, and try to combine within a same cluster certain habitats, taxa, contaminants, and pressures, among others. Considering a multitude of policy-driven targets and aims, research should also be directed to obtain some crucial knowledge needs for management (e.g., water quality, nature conservation, food quality and security, fisheries management, and aquaculture acts and directives). Despite its focused objectives, this knowledge can indeed increase our global knowledge on these issues, especially if integrated in meta-analyses studies and literature reviews.
